# Integrated analysis reveals crosstalk between pyroptosis and immune regulation in renal fibrosis

**DOI:** 10.3389/fimmu.2024.1247382

**Published:** 2024-01-26

**Authors:** Fengxia Bai, Longchao Han, Jifeng Yang, Yuxiu Liu, Xiangmeng Li, Yaqin Wang, Ruijian Jiang, Zhaomu Zeng, Yan Gao, Haisong Zhang

**Affiliations:** ^1^ School of Clinical Medicine, Hebei University, Affiliated Hospital of Hebei University, Baoding, China; ^2^ Hebei Provincial Key Laboratory of Skeletal Metabolic Physiology of Chronic Kidney Disease, Affiliated Hospital of Hebei University, Baoding, China; ^3^ Department of Gastrointestinal Oncology, Affiliated Xingtai People's Hospital of Hebei Medical University, Xingtai, China; ^4^ Department of Critical Care Medicine, The First Hospital of Hebei Medical University, Shijiazhuang, China; ^5^ Department of Neurosurgery, Jiangxi Provincial People’s Hospital, The First Affiliated Hospital of Nanchang Medical College, Nanchang, China

**Keywords:** renal fibrosis, pyroptosis, immune regulation, PYCARD, myofibroblasts, regulatory T cells (Tregs)

## Abstract

**Purpose:**

The pathogenesis of renal fibrosis (RF) involves intricate interactions between profibrotic processes and immune responses. This study aimed to explore the potential involvement of the pyroptosis signaling pathway in immune microenvironment regulation within the context of RF. Through comprehensive bioinformatics analysis and experimental validation, we investigated the influence of pyroptosis on the immune landscape in RF.

**Methods:**

We obtained RNA-seq datasets from Gene Expression Omnibus (GEO) databases and identified Pyroptosis-Associated Regulators (PARs) through literature reviews. Systematic evaluation of alterations in 27 PARs was performed in RF and normal kidney samples, followed by relevant functional analyses. Unsupervised cluster analysis revealed distinct pyroptosis modification patterns. Using single-sample gene set enrichment analysis (ssGSEA), we examined the correlation between pyroptosis and immune infiltration. Hub regulators were identified *via* weighted gene coexpression network analysis (WGCNA) and further validated in a single-cell RNA-seq dataset. We also established a unilateral ureteral obstruction-induced RF mouse model to verify the expression of key regulators at the mRNA and protein levels.

**Results:**

Our comprehensive analysis revealed altered expression of 19 PARs in RF samples compared to normal samples. Five hub regulators, namely PYCARD, CASP1, AIM2, NOD2, and CASP9, exhibited potential as biomarkers for RF. Based on these regulators, a classifier capable of distinguishing normal samples from RF samples was developed. Furthermore, we identified correlations between immune features and PARs expression, with PYCARD positively associated with regulatory T cells abundance in fibrotic tissues. Unsupervised clustering of RF samples yielded two distinct subtypes (Subtype A and Subtype B), with Subtype B characterized by active immune responses against RF. Subsequent WGCNA analysis identified PYCARD, CASP1, and NOD2 as hub PARs in the pyroptosis modification patterns. Single-cell level validation confirmed PYCARD expression in myofibroblasts, implicating its significance in the stress response of myofibroblasts to injury. *In vivo* experimental validation further demonstrated elevated PYCARD expression in RF, accompanied by infiltration of Foxp3^+^ regulatory T cells.

**Conclusions:**

Our findings suggest that pyroptosis plays a pivotal role in orchestrating the immune microenvironment of RF. This study provides valuable insights into the pathogenesis of RF and highlights potential targets for future therapeutic interventions.

## Introduction

Renal fibrosis (RF) represents a common end-stage consequence in various progressive kidney diseases, ultimately leading to end-stage renal disease (ESRD), regardless of the underlying cause. It is characterized by the disruption of typical kidney architecture, excessive accumulation of extracellular matrix (ECM), and activation of myofibroblasts (1). Research has shown that inflammation plays a crucial role in the pathogenesis of RF (2, 3). Due to chronic inflammation in the kidneys, fibroblasts transform into myofibroblasts in response to various pro-inflammatory factors and pro-fibrotic factors, exacerbating fibrosis. Epithelial-to-mesenchymal transformation (EMT) and endothelial-to-mesenchymal transformation (EndMT) are considered contributors to the origin of myofibroblasts. There are potential drugs evaluated for treating renal fibrosis, such as DPP-4 inhibitors, empagliflozin, glycolysis inhibitors, mineralocorticoid receptor antagonists, ARB/ACEI, and peptide AcSDKP (4–6). The DPP-4 inhibitor linagliptin can delay the progression of renal disease in non-diabetic rats with 5/6 nephrectomy (5). Sodium-glucose cotransporter 2 has beneficial effects on kidney injury, and dagliazine inhibits NLRP3 inflammasome activation, preventing kidney fibrosis (7). Mineralocorticoid receptor antagonists also show promise in reducing kidney inflammation and fibrosis (6). However, effective clinical therapies for RF remain limited. Therefore, gaining in-depth understanding of the molecular mechanisms regulating inflammation during the course of RF progression is crucial for developing effective treatment strategies to alleviate fibrosis.

Several signaling pathways, including TGF-β, Wnt, Notch, and Hedgehog pathways, have been identified to be activated in the development of RF (8). Additionally, new protective signaling molecules, such as endothelial glucocorticoid receptor (GR), FGFR1, and SIRT3, have been discovered. Endothelial GR regulates fibrogenesis by controlling canonical Wnt signaling and influencing fatty acid metabolism in the kidneys of diabetic mice (9) SIRT3 regulates mitochondrial protein deacetylation to slow down renal fibrosis (10). Pyroptosis is a form of proinflammatory programmed cell death mediated by caspases and gasdermins (GSDMs). Pyroptosis-associated signaling has been extensively studied. After activation by pathogens or danger signals, inflammatory caspases cleave GSDMs, releasing the N-terminal domain and forming membrane pores, leading to cell swelling, rupture, and the secretion of cell contents and proinflammatory cytokines (11, 12). Increasing evidence indicates that pyroptosis-induced inflammation and cellular injury are closely related to kidney diseases, exacerbating RF. NLRP3 is a critical node in the regulation of inflammation and pyroptosis. An activated NLRP3 inflammasome contributes to unilateral ureteral obstruction (UUO)-induced renal fibrosis, while gemigliptin has a renoprotective effect by attenuating NLRP3 activity (13). Subsequent studies showed that NLRP3 deletion restored kidney function, reduced pro-inflammatory cytokine release, and reversed mitochondrial dysfunction in a murine UUO model (14). In diabetes, caspase-1-mediated pyroptosis drives renal inflammation and fibrosis (15). Recent studies demonstrated that TNFα/Casp3/GSDME-mediated pyroptosis plays an important role in the renal tubule, triggering the release of HMGB1 and recruiting and activating immune cells, contributing to the progression of obstructive nephropathy and fibrosis (16). These findings indicate the involvement of pyroptosis in the occurrence and progression of RF. However, the underlying mechanisms of the interaction between pyroptosis and inflammation in RF are still poorly understood. Systematic exploration of pyroptosis-associated regulators (PARs) in the context of RF may provide valuable insights into RF treatment.

Over the past decade, RNA sequencing (RNA-seq) has emerged as a transformative technology in biological and medical research (17, 18). RNA-seq includes bulk RNA-seq and single-cell RNA-seq (scRNA-seq), proving instrumental in revealing cellular phenotypes and shedding light on the pathogenesis of complex diseases (19). In this study, we conducted a comprehensive assessment of Pyroptosis-Associated Regulators (PARs) in renal fibrosis. The relationship between pyroptosis and immune regulation in RF needs to be illustrated, leveraging bioinformatics approaches. Currently, molecular diagnostics for RF lack reliable molecular biomarkers. Moreover, investigating the role of pyroptosis and immune regulation in the progression of RF may potentially provide clues for identifying novel therapeutic targets.

## Materials and methods

### Datasets and data preprocessing

The following RNA-seq datasets were downloaded from the Gene Expression Omnibus (GEO) database: GSE22459, GSE76882, GSE135327, and GSE65326. The GSE22459, GSE76882, and GSE135327 datasets were normalized by log(x+1), and batch effects were removed with the combat function in the sva package (20), the resulting dataset was defined as the screening set, and it included 100 RF samples and 136 normal samples. For detailed information on the samples in each dataset, see **Supplementary File 1**. The GSE164647 dataset was downloaded in cloupe format as the scRNA-seq validation set (https://www.ncbi.nlm.nih.gov/geo/query/acc.cgi?acc=GSE164647), and Loupe Browser 6 software was used for data visualization. According to previous research on PARs (21), a total of 27 PARs were annotated in the final dataset.

### Screening for hub PARs

In the screening set, the differential expression of PARs was compared between RF and normal samples with the Wilcox test. The expression relationships among regulators in RF samples were evaluated by Spearman correlation analysis. Then, the Lasso regression algorithm was used to remove redundant genes (22). After redundant genes were removed, random forest and support vector machine (SVM) models (23) were constructed simultaneously, and the advantages and disadvantages of the two models were compared by calculating residuals. Regulators in the best model were selected as hub PARs, and they were included in the PCA model to calculate the pyroptosis score. The calibration curve and decision curve analysis (DCA) were used to evaluate the differential performance of the pyroptosis score. Finally, verification was performed in the same way with the validation set GSE65326.

### Identification of pyroptosis modification patterns

Unsupervised cluster analysis identified different pyroptosis modification patterns based on the expression of five hub PARs. A consensus clustering algorithm was used to evaluate the clustering number and robustness (24). Using the k-means clustering method, 100 iterative calculations were carried out (80% of the samples were used each time) to ensure stable clustering. The optimal number of clusters was determined on the basis of the clustering score of the CDF curve. PCA was used to verify the reliability of consensus clustering.

### Enrichment analysis

The gene sets “c2.cp.kegg.v7.4.symbols” and “c5.go.v7.4.symbols” were used to identify changes in biological signaling pathways (25). The expression matrix was transformed into a score matrix with the GSVA algorithm, the scores of biological signaling pathways were compared between different pyroptosis modification patterns with the limma package, and the threshold for the difference analysis was P <0.05. The limma package (26) was used to screen for genes with significant differences between different pyroptosis patterns. | |log2-fold change FC|>1 and adj.P.value <0.05 were selected as truncation criteria, and GO (Gene Ontology) functional enrichment and KEGG (Kyoto Encyclopedia of Genes and Genomes) pathway analysis were carried out with the clusterProfiler package (27).

### Difference and correlation analysis of immune characteristics

ssGSEA was used to estimate the number of specific infiltrating immune cells and the activity of specific immune responses and to explore the status of immune cells and immune-related pathways according to gene sets. The enrichment scores of immune cells and immune-related pathways among different groups were compared with the Kruskal-Wallis test. Spearman correlation analysis was used to determine the relevance of five hub PARs with respect to immune cells, immune response activity, and human leukocyte antigen (HLA) expression.

### WGCNA

In the weighted gene coexpression network analysis (WGCNA) (28), the top 25% of genes with maximum variance in the sample were used as inputs, and the topological calculation was carried out with a soft threshold of 1 to 25. According to the optimal soft threshold, the relation matrix was transformed into an adjacent matrix and then transformed into a topological overlap matrix (TOM), and average link hierarchical clustering was performed. The relevant modules were classified according to the TOM, and the number of genes in each module was not less than 20, merging of similar modules was then performed. Finally, the correlations between the merged modules and different pyroptosis modification subtypes were calculated.

### UUO-induced RF mouse model

All animal care procedures and animal studies were approved by the Animal Welfare and Ethical Committee of Hebei University and followed the Chinese Guidelines on the Care and Use of Laboratory Animals. UUO surgery was carried out by left ureteral ligation as previously described (29). Briefly, 10- to 12-week-old male C57BL/6 mice were anaesthetized with 1.5% isoflurane. Complete ureteral obstruction was performed by double ligation of the left ureter with 4-0 silk sutures tied just distal to the renal pelvis. The left ureters were identified but not ligated in sham-operated mice. Sham-operated kidneys were used as a control. At 7 or 14 days after surgery, the kidneys were harvested for RNA, protein and histologic analysis.

### Immunohistochemistry and immunofluorescence staining

The kidney tissues were fixed in 4% paraformaldehyde, embedded in paraffin, and sliced into 4μm thick sections. Histological staining was performed by using standard procedures. Hematoxylin and eosin (HE) staining was used for observing tubular injury. Collagen accumulation was determined by Masson trichrome staining and Sirius Red staining. Immunohistochemistry (IHC) was performed by routine protocols. Briefly, after deparaffinization and rehydration, the kidney sections were subjected to microwave antigen retrieval using EDTA buffer (pH 9.0) (ZSGB-Bio, Beijing, China), followed by blocking of endogenous peroxidase and nonspecific protein binding sites. Next, the tissue sections were incubated overnight in the corresponding primary antibodies at 4°C. Primary antibodies for IHC were: α-SMA (1:80, ab124964, Abcam), PYCARD (1:80, 67824, Cell Signaling Technology), and Foxp3 (1:100, ab215206, Abcam), E-cadherin (1:200, 20874-1-AP, Proteintech), Fibronectin (1:200, 66042-1-Ig, Proteintech), and CD31 (1:250, 28083-1-AP, Proteintech). The sections were then washed three times with PBS the following day and incubated with a horseradish peroxidase (HRP)-conjugated secondary antibody. Finally, the target signal was developed utilizing DAB solution, and the sections were stained with hematoxylin. Immunofluorescence staining was performed with paraffin sections. The kidney sections were incubated overnight at 4°C with different primary antibodies: PYCARD (1:80, 67824, Cell Signaling Technology), α-SMA (1:50, 67735-1-Ig, Proteintech), and Foxp3(1:80, FJK-16s, eBioscience). Alexa Fluor^®^488 and Alexa Fluor^®^ 594 labeled secondary antibodies were used as a detection antibody. DAPI was used to stain the nuclei. Images were captured by a light microscope (model BX43, Olympus, Japan) and fluorescence microscope (model IX71, Olympus, Japan), quantified with Image J software.

### Real-time quantitative polymerase chain reaction

Total RNA was extracted from mouse kidney tissues using the Eastep^®^ Super Total RNA Extraction Kit (LS1040, Shanghai Promega) based on the manufacturer’s instructions. The RNA was then reverse transcribed into complementary DNA (cDNA) using a HiScript III RT SuperMix for qPCR (+gDNA wiper) Kit (R323, Vazyme Biotech, Nanjing, China). RT-qPCR was performed in the LC96 real-time PCR system (Roche) using ChamQ Universal SYBR qPCR Master Mix (Q711, Vazyme Biotech) and gene-specific primers. The PCR conditions were as follows: predenaturation at 95°C for 30 s, denaturation at 95°C for 10 s, and annealing at 60°C for 30 s, with a total of 40 cycles. Glyceraldehyde 3-phosphate dehydrogenase (GAPDH) was utilized as an internal reference to normalize the mRNA expression levels. The final data were calculated using the 2^-ΔΔCT^ formula. All primer sequences used are listed in **Supplementary Table 1**.

### Western blot analysis

Protein was extracted from mouse kidney tissues using RIPA lysis buffer (Solarbio, R0010). Equal amounts of protein were separated by 12.5% sodium dodecyl sulfate-polyacrylamide gel electrophoresis (SDS-PAGE) and then transferred to 0.45 μm polyvinylidene difluoride (PVDF) membranes. The membranes were blocked with 5% fat-free milk in Tris-buffered saline with Tween 20 (TBST) for 2 h at room temperature and then incubated overnight at 4°C with a primary antibody, followed by incubation with the HRP-conjugated secondary antibody (1:20000, AB0101, Abways) for 1 h at room temperature. The following primary antibodies were used: α-SMA (ab124964, Abcam, 1:30000), PYCARD (67824, CST, 1:1000), Glucocorticoid receptor (1:1000, CY6612, Abways), SIRT3 (10099-1-AP, Proteintech, 1:3000), and GAPDH (10494-1-AP, Proteintech, 1:30000). The protein blots were visualized with ECL.

### Statistical analysis of experimental data

All statistical analyses were performed with GraphPad Prism software version 8.0 (GraphPad Software, San Diego, CA, USA). Differences between experimental groups were assessed by one-way analysis of variance (ANOVA). Data are presented as the means±SEMs. P values <0.05 were considered statistically significant.

## Results

### Expression landscape of PARs among different samples

**Figure 1:**
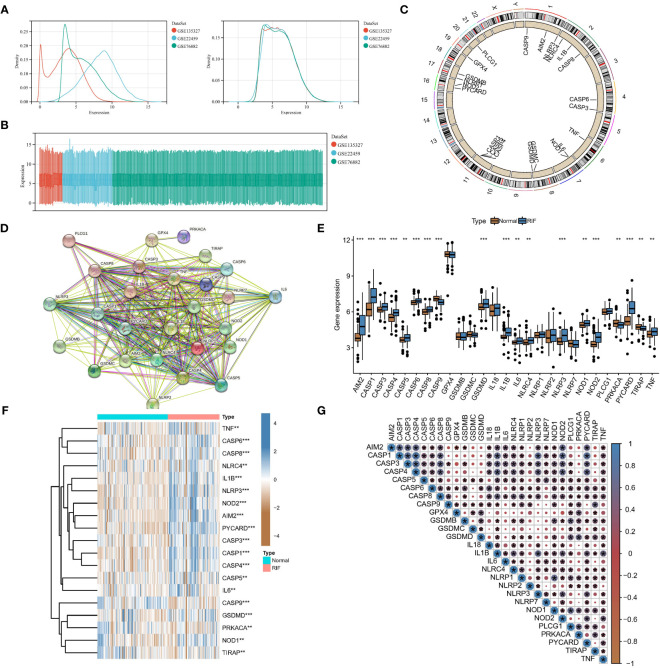
Expression landscape of PARs in RF. Eliminating data heterogeneity in the expression density plot **(A)** and expression box plot **(B)**. **(C)** Chromosomal locations of the 27 PARs. **(D)** The protein-protein interactions among pyroptosis regulators. **(E)** The box plot shows the expression of 19 regulators in RF compared to healthy samples. **(F)** The heatmap shows the expression status of 19 regulators in RF compared to healthy samples. **(G)** Interrelationships of the expression levels of PARs in RF samples. *P < 0.05, **P < 0.01, and ***P < 0.001.

GSE22459, GSE76882, and GSE135327 were normalized by log(x+1), and batch correction was performed with the combat function. The expression density plot (**Figure 1A**) and expression box plot (**Figure 1B**) showed that heterogeneity was eliminated in the corrected combined data. **Figure 1C** shows the chromosomal locations of 27 PARs: AIM2, CASP1, CASP3, CASP4, CASP5, CASP6, CASP8, CASP9, GPX4, GSDMB, GSDMC, GSDMD, IL18, IL1B, IL6, NLRC4, NLRP1, NLRP2, NLRP3, NLRP7, NOD1, NOD2, PLCG1, PRKACA, PYCARD, TIRAP, and TNF. The regulatory interactions of PARs are shown in protein-protein interaction (PPI) networks (**Figure 1D**), and we noted that the regulators are very closely related and usually function as a complex. The Wilcoxon test showed significant differences in the expression of 19 regulators in different samples (**Figures 1E, F**). Subsequently, the correlations among the expression levels of different regulators were explored in RF samples (**Figure 1G**), and we found that most of the pyroptosis regulators were closely related in RF samples, which further confirmed the results obtained with our PPI network.

### PARs can be used as potential biomarkers for RF

**Figure 2:**
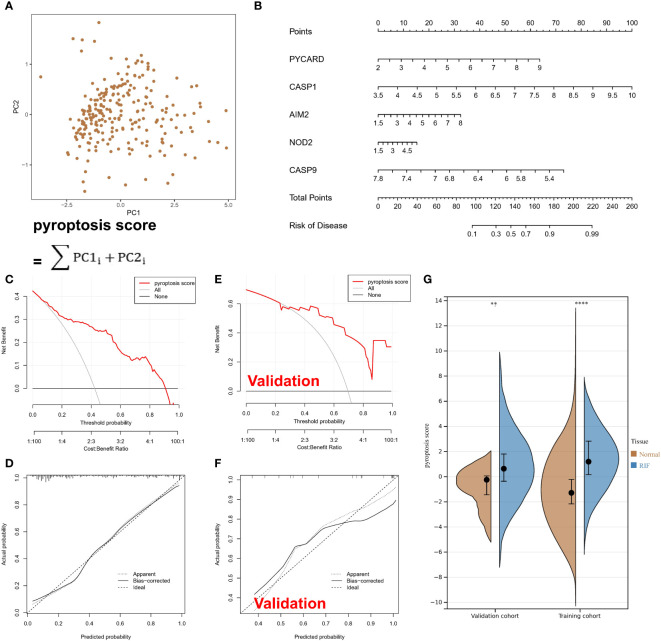
Pyroptosis-associated regulators can be used to classify normal and RF samples. **(A)** Calculated pyroptosis score for each sample with the PCA model. **(B)** A nomogram. **(C)** In the training set, the red line remained above the grey line in the DCA curve. **(D)** In the training set, the calibration curve shows the predicted probability for the pyroptosis score and actual probability. **(E)** In the validation set, the red line remained above the grey line in the DCA curve. **(F)** In the validation set, the calibration curve shows the predicted probability for the pyroptosis score and actual probability. **(G)** The violin plot shows higher scores for RF samples. **P < 0.01, and ****P < 0.0001.

To study the contribution of PARs to the pathogenesis of RF, 27 regulators were analysed by LASSO regression with feature selection and dimensionality reduction to exclude redundant genes (**Supplementary Figure 1A**). Finally, 9 regulators were used for subsequent analysis. Then, the residuals of different machine learning models were compared (**Supplementary Figure 1B**), and the gene model screened with the random forest model had the smallest residuals among different reverse cumulative distributions. The genes with gene importance greater than 10 in the random forest model included PYCARD, CASP1, AIM2, NOD2, and CASP9 (**Supplementary Figures 1C, D**). However, unfortunately, with the SVM model, there was not a good gene elimination effect (**Supplementary Figure 1E**), 9 regulators were identified, and the RSME was the smallest. Therefore, the SVM model was considered the best model, with PYCARD, CASP1, AIM2, NOD2, and CASP9 being considered the hub PARs. As shown in **Figure 2A**, the pyroptosis score for each sample was calculated to quantify the pyroptosis modification of each sample based on the 5 hub regulatory factors in the PCA model. A nomogram was constructed for clinical practice (**Figure 2B**). In addition, the red line in the DCA curve remained above the grey line in the training set (**Figures 2C, D**) and validation set (**Figures 2E, F**), indicating that making decisions based on the pyroptosis score may benefit RF patients. The calibration curve likewise showed that the prediction made according to the pyroptosis score was accurate. Moreover, based on the pyroptosis scores from the PCA algorithm, we found that the RF samples had a higher score than the normal samples (**Figure 2G**).

### PARs are associated with immune responses in RF tissue

**Figure 3:**
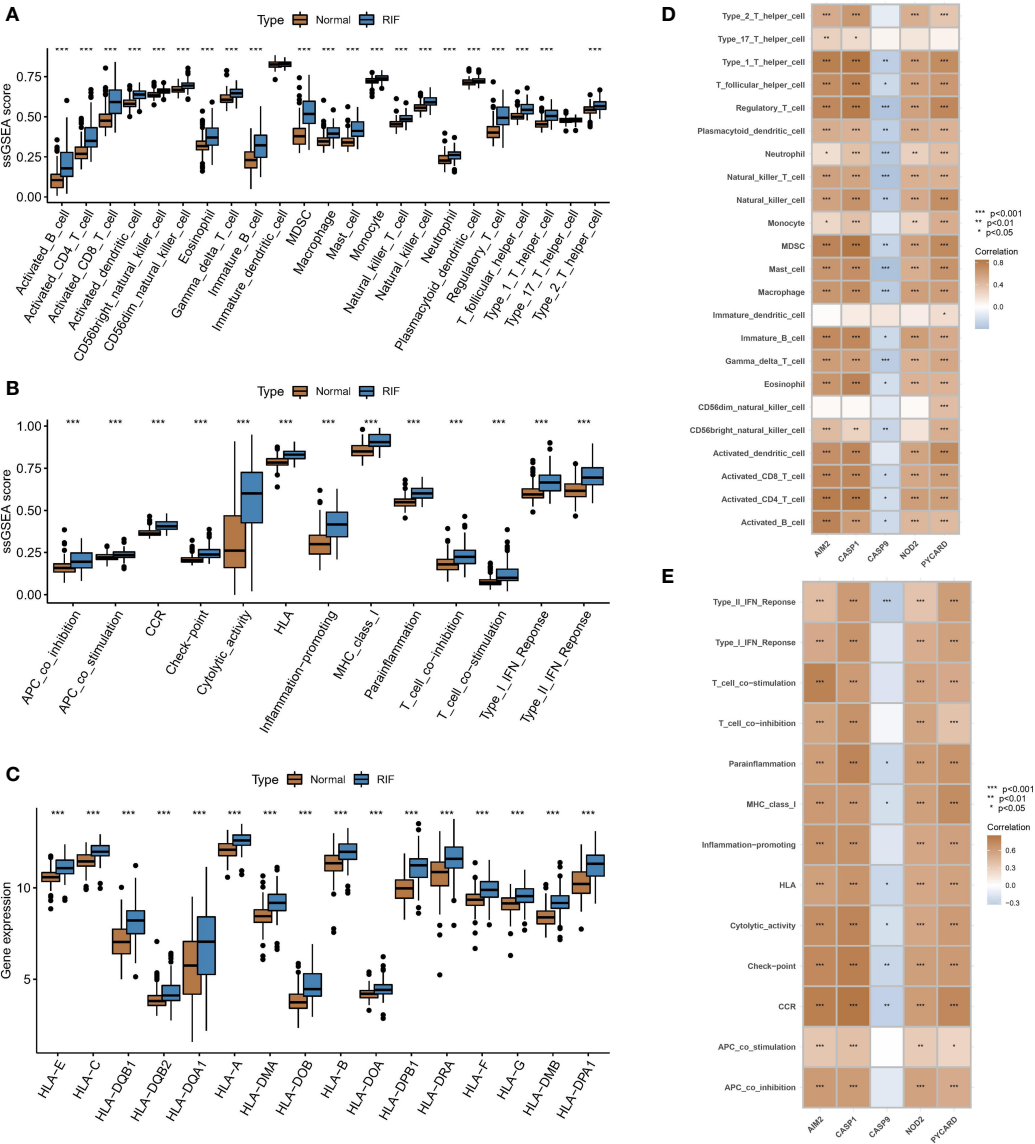
Associations between PARs and immune responses in RF. Differences in **(A)** the abundance of infiltrating cells, **(B)** immune function, and **(C)** HLA expression in the immune microenvironment between healthy samples and RF samples. Results of correlation analysis of five hub regulators with **(D)** immune cells and **(E)** immune function in fibrosis samples. *P < 0.05, **P < 0.01, and ***P < 0.001.

**Figure 4:**
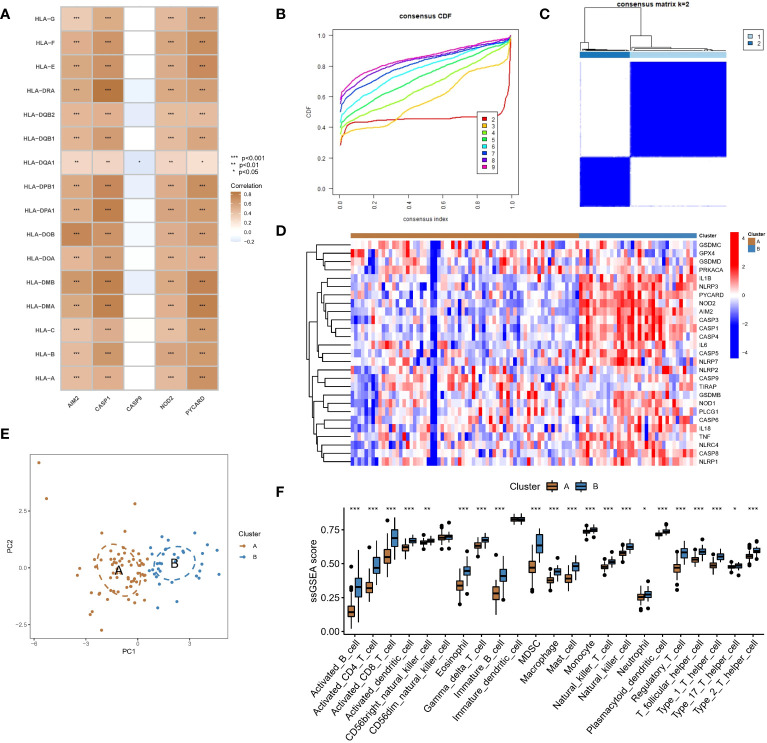
Identification of two different pyroptosis modification pattern subtypes in RF. **(A)** Results of correlation analysis of five hub regulators with HLA expression in RF samples. **(B)** A consensus clustering cumulative distribution function (CDF) with k =2-9 is shown. **(C)** Heatmap of the matrix of cooccurrence proportions for RF samples. **(D)** Heatmap of the expression status of PARs in the two pyroptosis modification patterns. **(E)** Principal component analysis (PCA) of the transcriptome profiles of the two pyroptosis modification patterns. **(F)** Differences in the abundance of infiltrating immunocytes between the two pyroptosis modification patterns. *P < 0.05, **P < 0.01, and ***P < 0.001.

Differential expression analysis revealed differences in the abundance of infiltrating cells in the immune microenvironment, immune function, and HLA expression between healthy samples and RF samples. Compared with those in normal kidney samples, the abundances of most immune killing cells, such as macrophages and activated T cells were altered in RF samples (**Figure 3A**). The significant activation of inflammatory response pathways in RF samples may indicate that such pathways are involved in the development of RF (**Figure 3B**). In addition, most HLA molecules were significantly upregulated in RF samples (**Figure 3C**). To study the relationship between pyroptosis regulators and the immune microenvironment, correlation analysis of the five hub regulators mentioned above and infiltrating immunocytes, immune-related signaling pathways, and HLA expression was analysed. The correlation analysis showed that the expression levels of most of the hub regulators were positively correlated with the abundance of many immunocytes in RF samples, while only CASP9 expression was negatively correlated with the abundance of most immunocytes (**Figure 3D**). In particular, PYCARD expression was positively correlated with the abundance of regulatory T cells (Tregs) in fibrotic tissues. In terms of immune function (**Figure 3E**), HLA expression (**Figure 4A**) showed similar results. The above results indicate that hub PARs play important roles in the immune microenvironment in RF.

### PAR-mediated pyroptosis modification patterns in RF

We conducted an unsupervised consensus clustering analysis of 100 RF samples based on the expression of hub regulators (**Figure 4B**). Two different pyroptosis modification subtypes were identified (**Figure 4C**), and the heatmap showed that most regulators positively correlated with immune pathways were upregulated in subtype B, while CASP9, which was negatively correlated with immune pathways, was downregulated (**Figure 4D**). PCA showed that RF patients could be divided into two groups based on the expression of PARs (**Figure 4E**). To identify the differences in immune microenvironmental characteristics between these different pyroptosis modification patterns, we evaluated differences in infiltrating immune cells and immune functions. Compared with subtype A, subtype B had a relatively activated immune microenvironment (**Figure 4F**). In terms of immune function, the immune response of subtype B was more active (**Supplementary Figure 2A**). In addition, the expression of different HLAs differed between modification patterns (**Supplementary Figure 2B**), and subtype B was again more active. The above results again proved that pyroptosis modification patterns play an important role in regulating the formation of different immune microenvironments in RF, with subtype B representing a more active immune microenvironment.

### Biological characteristics of different pyroptosis modification patterns

To investigate the biological characteristics of different pyroptosis modification patterns, we compared their associated KEGG pathways and applied GSVA enrichment analysis to evaluate the activation status of biological pathways. The top 10 differential pathways are shown in the heatmap, and natural killer cell-mediated cytotoxicity, primary immunodeficiency, apoptosis, and other signaling pathways were significantly enriched in subtype B compared with subtype A (**Supplementary Figure 3A**). In addition, regarding GO terms, most of the pathways in subtype B were more active than those in subtype A, such as the nucleotide-binding domain leucine-rich repeat containing receptor signaling pathway, T-cell differentiation in thymus, and T-cell differentiation (**Supplementary Figure 3B**). In addition, a total of 280 differentially expressed genes (DEGs) between the modification patterns were identified (**Supplementary Figure 3C**), and subsequent enrichment analysis showed that the screened DEGs were significantly correlated with signaling pathways of various immune cells in GO analysis (**Supplementary Figure 3D**). Similarly, KEGG enrichment analysis showed that these DEGs were mainly involved in a variety of immune regulation processes (**Supplementary Figure 3E**).

### Identifying hub regulators of the pyroptosis modification patterns through WGCNA

**Figure 5:**
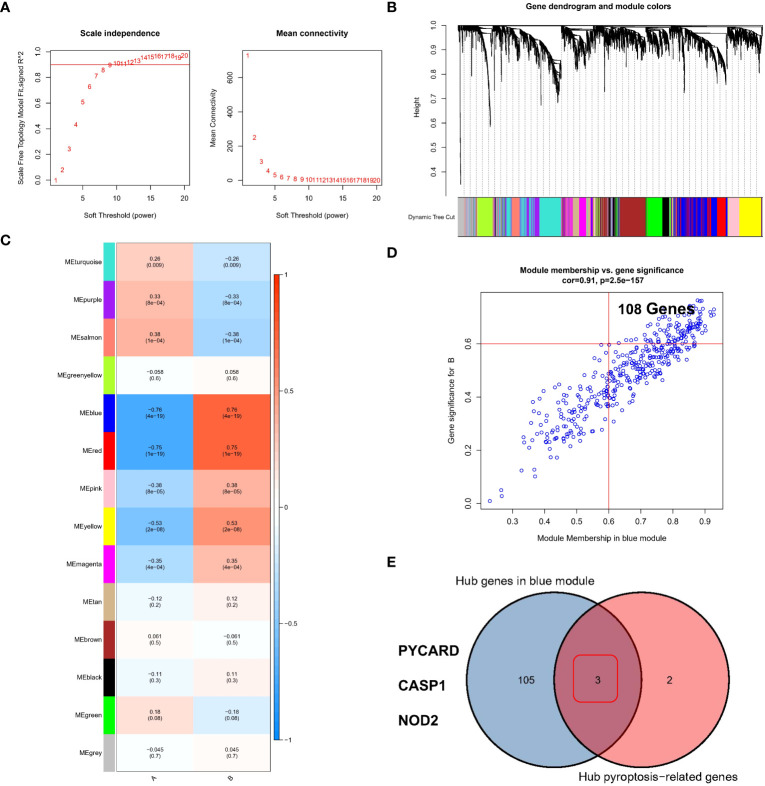
Identification of the hub regulators involved in different pyroptosis modification patterns in RF. **(A)** Analysis of the scale-free ft index and mean connectivity for various soft-thresholding powers. **(B)** Gene tree diagram. **(C)** The heatmap shows 14 gene modules in the different pyroptosis modification patterns. **(D)** Gene significance (GS) scatter plot of pyroptosis modification pattern B and module member (MM) in the blue module. **(E)** Three genes were considered to be hub PARs in different pyroptosis modification patterns.

We used the WGCNA method and inputted the top 25% of genes with the largest variance in the sample. After removing the outlier samples, we identified gene modules of different colors (**Figure 5B**) based on the best soft threshold of 8 (**Figure 5A**) and clustered similar modules. Finally, we identified 14 different gene modules (**Figure 5C**), and the different modification patterns were correlated with the genes in each module. Among the investigated correlations, the correlation between the blue module and different modification patterns was the strongest (r=0.76/-0.76). Then, the GS and MM for the blue module were calculated, and 108 genes that may be related to the modification patterns were identified using GS>0.6 and MM>0.6 as thresholds (**Figure 5D**). Finally, PYCARD, CASP1, and NOD2 (**Figure 5E**), which overlapped with the hub pyroptosis regulators, were identified as potential hub genes driving the progression from normal tissue to RF, as well as different pyroptosis modification patterns.

### Analysis of the expression of PYCARD, CASP1 and NOD2 at the single-cell level

**Figure 6:**
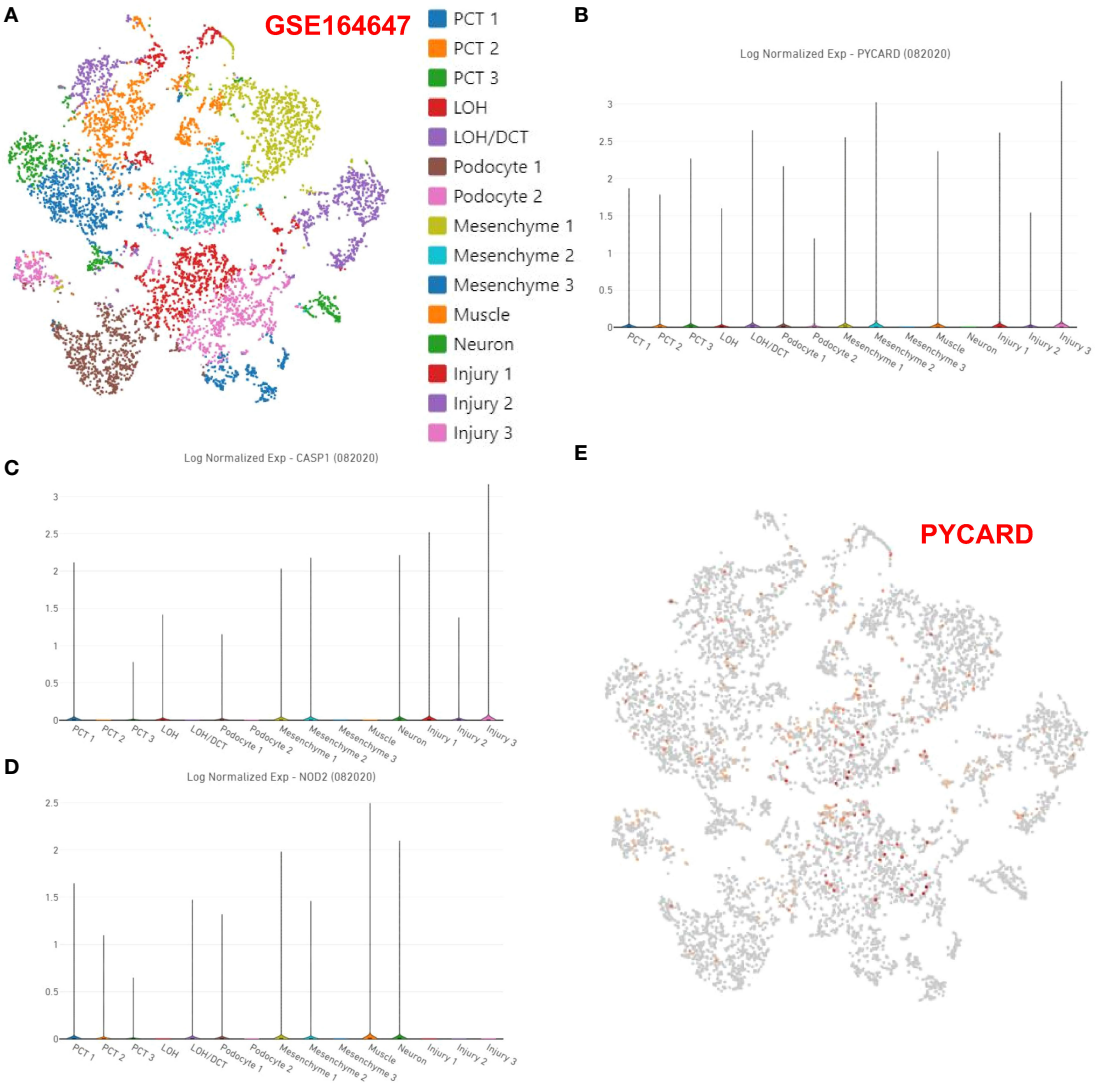
Expression of PYCARD, CASP1, and NOD2 at the single-cell level. **(A)** In Loupe Browser 6 software, 15 potential subgroups were acquired in the RF single-cell samples. The violin plot shows the distribution of **(B)** PYCARD, **(C)** CASP1, and **(D)** NOD2. **(E)** The expression of PYCARD is marked with red dots in the single-cell samples.

As described in the Methods section, in RF single-cell samples, on the basis of previous studies reporting 15 potential subgroups (**Figure 6A**), PCT1/2/3, LOH, LOH/DCT, Podocyte1/2, Mesenchyme1/2/3, Muscle, Neuron and injury1/2/3 were obtained by screening for highly variable genes by PCA in Loupe Browser 6 software. The violin plot showed the distribution of the PYCARD (**Figure 6B**), CASP1 (**Figure 6C**), and NOD2 (**Figure 6D**) genes. Unfortunately, only PYCARD was widely distributed and highly expressed in single-cell subgroups, with its main distribution site in mesenchyme cluster 1 and cluster 2, and injury clusters (**Figure 6E**). This finding suggests that PYCARD may play a critical role in myofibroblast activation after injury.

### Validation of the expression levels of PYCARD and Foxp3 in a mouse RF model

**Figure 7:**
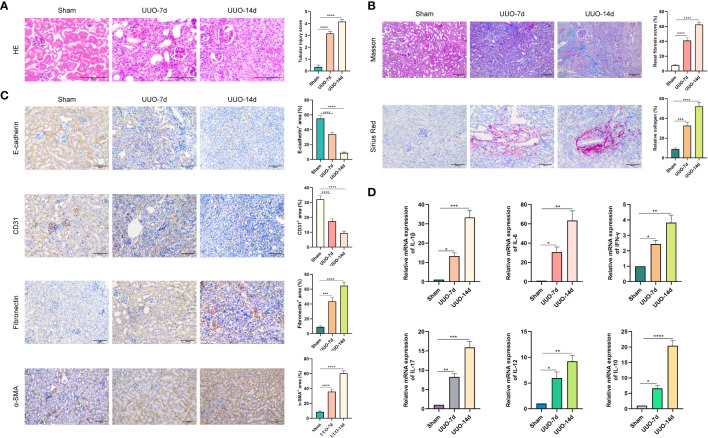
Renal fibrosis in UUO mouse models. UUO was performed on the left ureter to induce RF. Kidneys were isolated from sham-operated mice or from mice subjected to UUO on days 7 and 14 after UUO surgery. **(A)** Kidney tissues were stained with HE. Representative images are shown. Scale bar=100μm. Quantitative analysis of tubular injury score by HE staining. **(B)** Kidney tissues were stained with Masson trichrome and Sirius Red. Representative images are shown. Scale bar=100μm. Quantitative analysis of fibrotic area and collagen deposits evaluated by Masson trichrome and Sirius Red staining. **(C)** Representative images of the epithelial cell marker E-cadherin, the endothelial cell marker CD31, and the mesenchymal cell markers Fibronectin and α-SMA. Scale bar=100μm. Quantification of E-cadherin, CD31, Fibronectin and α-SMA expression by immunohistochemical staining. **(D)** The expression levels of IL-1β, IL-6, IFN-γ, IL-17, IL-12, and IL-10 in kidney tissues were quantified using RT-qPCR analysis. *P < 0.05, **P < 0.01, ***P < 0.001, and ****P < 0.0001.

**Figure 8:**
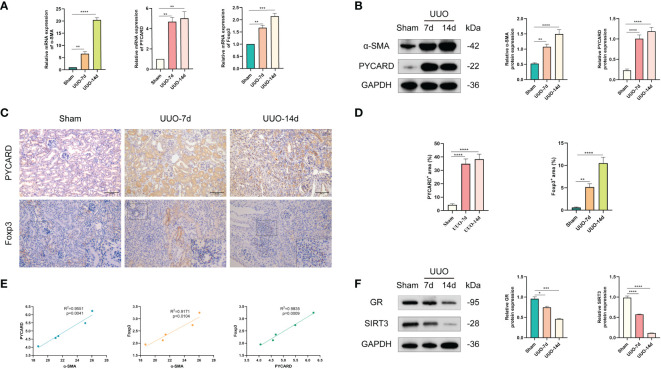
Validation of the expression levels of PYCARD and Foxp3 in sham-operated and UUO-induced RF mouse kidney tissues. **(A)** The expression levels of α-SMA, PYCARD, and Foxp3 in kidney tissues were quantified using RT-qPCR analysis. **(B)** Western blot analysis of α-SMA and PYCARD protein expression in kidney tissue. Kidney tissue lysates were isolated on days 7 or 14 after sham or UUO surgery. **(C)** Representative images of immunohistochemical staining for PYCARD and Foxp3 in kidney sections from mice. Scale bar=100μm. **(D)** Quantification of PYCARD and Foxp3 expression by immunohistochemical staining. **(E)** The relationships between the expression levels of α-SMA, PYCARD, and Foxp3 in RF samples are shown in the scatter plots. **(F)** Western blot analysis of glucocorticoid receptor (GR) and SIRT3 protein expression in kidney tissue. *P < 0.05, **P < 0.01, ***P < 0.001, and ****P < 0.0001.

According to the bioinformatics analysis results for the RNA-seq datasets of RF samples above, we found a positive correlation between PYCARD expression and the abundance of Tregs (**Figure 3D**). Tregs are a T-cell subset that controls the autoimmune response in the body. The transcription factor forkhead box protein 3 (Foxp3) is regarded as a characteristic marker of Tregs. To verify the expression of PYCARD and Foxp3 on renal fibrosis *in vivo*, we first established a mouse model of renal fibrosis induced by UUO. We observed increasing kidney damage in UUO mice on post-operative days 7 and 14 compared to sham-operated mice, as evidenced by interstitial injuries revealed by HE staining (**Figure 7A**), and collagen deposition shown by Masson trichrome staining and Sirius red staining (**Figure 7B**). In the UUO group, the decrease in the epithelial cell marker E-cadherin with the endothelial cell marker CD31 and the increase in the mesenchymal cell markers Fibronectin and α-SMA indicated that that the EMT and EndMT processes were occurring (**Figure 7C**). Concomitantly, the levels of IL-1β, IL-6, IFN-γ, IL-17, IL-12, and IL-10 were increased significantly in the renal tissues of UUO mice, suggesting the development of inflammation in kidneys (**Figure 7D**). In addition, we further detected the expression of the fibrosis-related protein α-SMA by RT-qPCR and Western blot (**Figures 8A, B**). Taken together, these results indicated that the UUO model was successful.

The expression of PYCARD and Foxp3 in healthy mouse kidney tissues and UUO-induced RF kidney tissues was further validated *in vivo*. As anticipated, mice subjected to UUO exhibited elevated mRNA expression of PYCARD and Foxp3 (**Figure 8A**) compared to mice subjected to sham surgery, aligning with the findings of bioinformatics analysis. Western blot analysis on days 7 and 14 of UUO confirmed the increased protein expression of PYCARD (**Figure 8B**). The immunohistochemical analysis of PYCARD and Foxp3 expression in renal tissue sections (**Figures 8C, D**) demonstrated a notable increase in PYCARD expression and the presence of Foxp3-positive cells following UUO induction. Furthermore, correlation analysis based on RT-qPCR Data revealed a positive association between PYCARD and α-SMA expression, as well as between Foxp3 and α-SMA expression (**Figure 8E**). Our findings indicate that PYCARD exhibited high expression levels in the RF group and exhibited a positive correlation with the infiltration of Foxp3^+^ Tregs. In addition, the involvement of glucocorticoid receptor (GR) and SIRT3 as crucial anti-inflammatory factors in UUO mouse models prompted us to investigate the protein expression of GR and SIRT3 in the kidney using Western blot analysis (**Figure 8F**). Our findings revealed a significant decrease in the expression levels of GR and SIRT3 in the UUO group compared to the control group.

**Figure 9:**
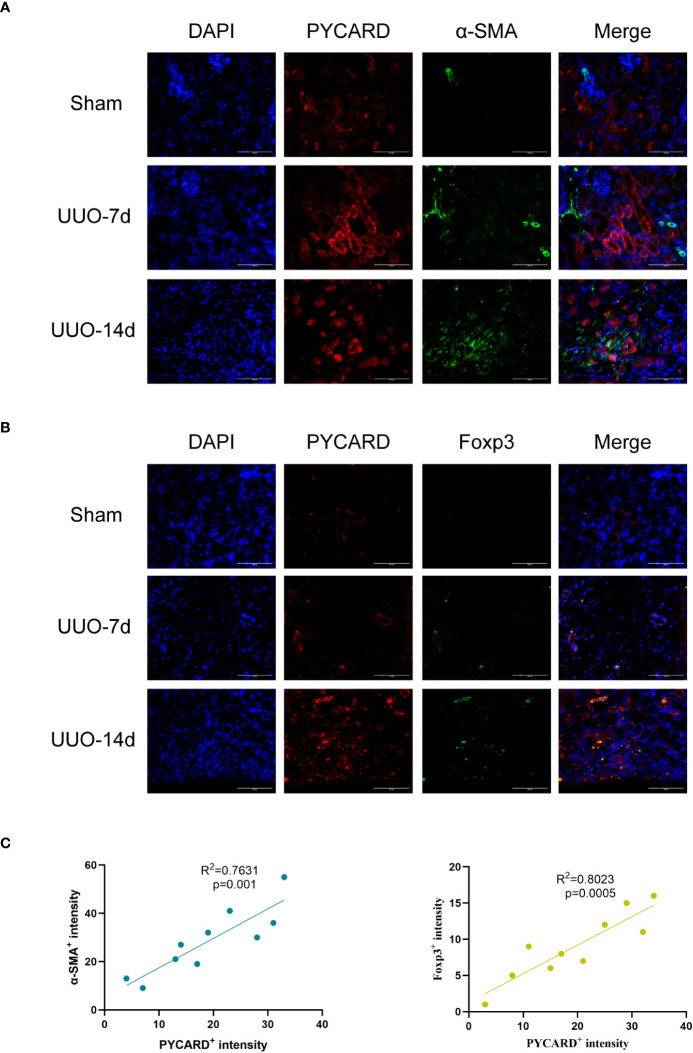
Immunofluorescence validation experiments and analysis in RF animal model. **(A)** Representative images of Immunofluorescence staining for PYCARD (red), α-SMA (green), and the nucleus (DAPI; blue) in kidney sections from mice. Scale bar=100μm. **(B)** Representative images of Immunofluorescence staining for PYCARD (red), Foxp3 (green), and the nucleus (DAPI; blue) in kidney sections from mice. Scale bar=100μm. **(C)** The relationships between PYCARD^+^ intensity and both α-SMA^+^ and Foxp3^+^ intensity in kidney samples. The PYCARD^+^, α-SMA^+^ and Foxp3^+^ intensity was assessed in 10 fields per slide at × 400 magnification.

To enhance our understanding of the spatial association between PYCARD and α-SMA, as well as PYCARD and Foxp3, we conducted additional immunofluorescence validation experiments on samples obtained from the RF animal model. In these experiments, immunofluorescence co-staining of PYCARD with α-SMA and PYCARD with Foxp3 proteins was conducted in the RF samples (**Figures 9A, B**). Subsequently, the obtained immunofluorescence results were analyzed and quantified (**Figure 9C**). Notably, a significant positive correlation between PYCARD expression and both α-SMA and Foxp3 was observed. These findings are highly consistent with our bioinformatics analysis, providing robust validation of the involvement of pyroptosis and immune regulation in RF.

## Discussion

The role of pyroptosis and immune responses in RF is not yet fully understood. This study systematically explored the differential expression of 27 PARs in RF and normal kidney samples and conducted relevant functional analyses. Among these factors, PYCARD, CASP1, AIM2, NOD2, and CASP9 were identified as hub PARs, which may potentially serve as biomarkers for RF. We developed a classifier based on five hub PARs. The classifier performed well in distinguishing between healthy and RF samples, revealing that PARs do indeed play a critical role in RF. In addition, differences in the abundance of immune cells in the immune microenvironment do exist between healthy and RF samples. PYCARD was strongly positively correlated with Tregs abundance. To investigate pyroptosis modification patterns in RF, two distinct RF subtypes were identified. Pyroptosis modification patterns play an important role in regulating the formation of different immune microenvironments in RF, with subtype B representing a more active immune response. PYCARD, CASP1, and NOD2 may be potential hub genes that drive the progression from normal tissue to RF. Then, PYCARD, CASP1, and NOD2 were further validated at the single-cell level. We found that PYCARD was highly expressed in mesenchyme cluster 1 and cluster 2, as well as injury clusters. It may play a key role in myofibroblast activation after injury. The results showed that PYCARD was highly expressed in the fibrotic kidney and positively correlated with the infiltration of Tregs. Finally, we conducted a validation experiment in a mouse RF model. Histologic and RT-qPCR analysis of kidneys suggests the development of inflammation, EMT, and EndMT in kidneys. Western blotting and IHC revealed a significant increase in PYCARD expression and Foxp3-positive cells after UUO induction. Conversely, we observed a decrease in GR, accompanied by a reduction in SIRT3 in UUO. Additionally, immunofluorescence analysis demonstrated a positive correlation between PYCARD expression and both α-SMA and Foxp3. Bioinformatics and *in vivo* experiments revealed that pyroptosis exerts an important regulatory effect on the immune microenvironment in the context of RF. This study provides important insights into pyroptosis in RF to facilitate the characterization of pyroptosis mechanisms and immune characteristics in RF by other researchers.

The best approach that clinicians can use to assess the degree of RF is kidney biopsy, which is an invasive procedure used to monitor the progression of the disease in specific circumstances. Thus, the identification of noninvasive RF biomarkers would be of fundamental importance. Researchers have identified MCP-1 (monocyte chemoattractant protein-1) (30), KIM-1 (kidney injury molecule-1) (31), MMP-7 (matrix metalloproteinase-7) (32), heparanase-1 (33), cadherin-11, SMOC2 (sparc-related modular calcium binding protein-2), and PEDF (pigment epithelium-derived factor) (34) as promising noninvasive biomarkers of kidney fibrosis. Despite numerous studies, no effective biomarkers are routinely employed for RF in clinical practice. In our research, we found differences in the expression of PYCARD, CASP1, AIM2, NOD2, and CASP9 between healthy and RF samples, and these factors may be potential diagnostic biomarkers for RF patients. However, the use of a single gene as a potential biomarker of RF has limitations because we cannot rule out high expression of the gene in other diseases. Therefore, in this study, we established a PCA diagnostic model based on the five hub pyroptosis regulators, PYCARD, CASP1, AIM2, NOD2, and CASP9, and calculated the pyroptosis score for each sample. After pyroptosis modification was quantified for each sample, higher scores were obtained for RF samples than for normal samples. The PCA model had a strong predictive ability for patients with RF. In conclusion, the construction of this disease prediction model score based on multiple genes as a diagnostic tool may lead to additional diagnostic advances in the future.

Five hub PARs, namely, PYCARD, CASP1, NOD2, AIM2, and CASP9, have been previously reported to be associated with inflammation and fibrosis. PYCARD, which is commonly known as apoptosis-associated speck-like protein containing a caspase recruitment domain (ASC), is the adaptor protein of inflammasomes and plays an important role in inflammation and pyroptosis. PYCARD contains a caspase activation and recruitment domain (CARD) and a pyrin domain (PYD) (35). Recent evidence revealed a crucial role of PYCARD in renal injury after UUO, and PYCARD deficiency reduces renal inflammation and fibrosis (36). Similarly, pulmonary inflammation and fibrosis are efficiently ameliorated by inhibiting the NLRP3-PYCARD interaction and pyroptosis (37). Caspase-1 (CASP1) is a major effector protease of pyroptosis. Experimental studies have reported that the inflammasome-caspase-1 pathway contributes to the development of inflammation and fibrosis in the liver and lungs *via* bioactive IL-1β and IL-18 production (38, 39). NOD2 (nucleotide oligomerization domain 2) plays an important role in the host response to bacterial infection. NOD2-deficient mice are protected from cholestasis-induced liver injury and fibrosis (40). The activation of the AIM2 (Absent in melanoma 2) inflammasome by self and foreign double-stranded DNA (dsDNA) mediates pyroptosis (41). AIM2 is expressed in the kidney and regulates renal inflammation and fibrosis in mice after UUO (42). Caspase-9 (CASP9) is an initiator caspase in the apoptosis process and is regulated by several signaling pathways (43). The expression levels of CASP9 in the kidney were shown to correlate with fibrosis severity in FA and UUO fibrosis models and were used as a kidney disease risk gene (44). Therefore, previous studies on the functions of the five hub pyroptosis regulators identified here are consistent with the conclusions of our mechanistic analysis.

In our study, the results obtained at the single-cell level suggest that PYCARD is expressed in myofibroblasts and is important for the stress response of myofibroblasts in response to injury. Fibrosis is a process characterized by repeated injury. In response to injury, myofibroblasts express PYCARD to regulate inflammation and the immune response, which is related to the progression of fibrosis. In other words, the main gene expression change that occurs in myofibroblasts in response to injury is the expression of PYCARD, which is important for immune regulation during the process of fibrosis. Difference and correlation analysis of immune characteristics revealed the important role of PYCARD in the immune microenvironment of RF. In RF samples, PYCARD expression was positively correlated with the abundance of many immune cells, with similar results for immune function and HLA expression. Moreover, we found that PYCARD was positively correlated with Tregs abundance. Tregs play a pivotal role in the adaptive immune response. Foxp3 plays an important role in Tregs development and immune regulation. In this study, PYCARD expression and the abundance of Tregs were positively correlated and participated in various immune regulatory processes. *In vivo* validation experiments showed that PYCARD and Foxp3 were upregulated in fibrotic kidneys. In the present study, high expression of PYCARD promoted fibrosis, and Treg numbers were positively correlated with PYCARD, so it was speculated that Tregs might also promote fibrosis.

In recent years, the role of Tregs in the progression of fibrotic diseases has been widely studied, but the mechanism of their profibrotic or antifibrotic function has not been fully elucidated. Studies have reported that the proportion and absolute number of Tregs are significantly increased in idiopathic pulmonary fibrosis (IPF) patients (45). Another study showed that reversing Tregs differentiation by blocking the PD-1 pathway ameliorated pulmonary fibrosis (46). Tregs promote liver fibrosis by activating hepatic stellate cells through the TGF-β pathway (47). This evidence indicates a profibrotic role for Tregs. Conversely, significantly impaired Foxp3^+^ Tregs activity was observed in the peripheral blood and bronchoalveolar lavage in patients with IPF (48). In an obstructive jaundice mouse model, liver Tregs expansion suppresses the function of bulk liver T lymphocytes, which effectively limits liver injury and fibrosis (49). Previous studies have reported that increasing the renal infiltration of Foxp3^+^ Tregs with periodate-oxidized ATP attenuated renal ischaemia-reperfusion injury (IRI) and improved renal recovery (50). Recently, researchers have revealed the unexpectedly divergent roles of Tregs in repair and fibrosis following kidney injury *via* kidney immune cell landscaping and gene expression profiling. Single-cell RNA sequencing revealed late Tregs accumulation during fibrosis. However, prophylactic expansion of Tregs using IL-2c and IL-33 before an injury has a protective effect during kidney injury and fibrosis. Due to the existence of different inflammatory environments, Tregs can express different sets of response genes within the same tissue (51). It should be noted that the dual role of Tregs and their regulatory mechanisms in RF need to be studied systematically and in detail. Therefore, PYCARD and Foxp3^+^ Tregs may be involved in the progression of RF. The interaction between pyroptosis and the immune response plays an important role in the development of RF, but the specific mechanism still needs to be further studied.

In the current study, we systematically revealed the potential association between pyroptosis and immune responses in RF. A mouse UUO model was constructed to verify the expression of PYCARD and Foxp3 in the fibrotic kidney at the levels of mRNA transcription and protein translation *in vivo*. Consistent with the results of bioinformatics analysis, this study demonstrated that the expression of PYCARD and Foxp3 was upregulated in the kidney following UUO. Our findings establish an important link between pyroptosis and immune responses. These results may offer clues for new therapeutic strategies for RF. However, our study still has some limitations. The main conclusions were drawn from bioinformatics analysis of RNA-seq datasets and lack experimental validation. The specific mechanism by which pyroptosis-associated regulators affect the immune response in RF needs to be studied in detail. We believe that investigations of the molecular pathways that link pyroptosis and the immune microenvironment in RF will be important in the future.

## Conclusion

In summary, our study showed that pyroptosis associated regulators participate in the development of RF and that PYCARD, CASP1, NOD2, AIM2, and CASP9 may be potential markers of RF. Pyroptosis associated regulators are related to alterations in immune cells and the immune response in RF. In addition, PYCARD is expressed by myofibroblasts, and it may be involved in immune regulation; this suggests a role of myofibroblasts in fibrosis immune regulation. Our work provides novel ideas for understanding the pathogenesis of RF.

## Data availability statement

The original codes used for the analyses presented in the study are publicly available. This data can be found here: https://github.com/yxyzhanghaisong/renal-fibrosis-and-pyroptosis/blob/main/core_code.r.

## Ethics statement

The animal study was approved by the Animal Welfare and Ethical Committee of Hebei University (Approval No. IACUC-2021003SM). The study was conducted in accordance with the local legislation and institutional requirements.

## Author contributions

FB, YG and HZ supervised the project and designed this study. FB, YL, JY and XL organized the public data and prepared all the figures and tables. FB, LH, JY and YW conducted the data analysis. FB, LH and RJ drafted the manuscript. FB, LH, JY, ZZ, YG and HZ revised the manuscript. All authors contributed to the article and approved the submitted version.
