# Supplementary material for: Integrated analysis reveals crosstalk between pyroptosis and immune regulation in renal fibrosis
Source: Front Immunol. 2024 Jan 26;15:1247382. doi: 10.3389/fimmu.2024.1247382 (PMC10853448; doi:10.3389/fimmu.2024.1247382)
Supplement: Supplementary file 1 [file DataSheet_1.docx]

***Supplementary Material***

**Integrated analysis reveals crosstalk between** **pyroptosis and immune regulation in renal fibrosis**

Fengxia Bai^1,2^, Yuxiu Liu^1,2^, Jifeng Yang^1,2^, Xiangmeng Li^1,2^, Yaqin Wang^3^, Longchao Han^4^, Ruijian Jiang^1,2^, Yan Gao^1,2^, Haisong Zhang^1,2*^

^1^School of Clinical Medicine, Hebei University, Affiliated Hospital of Hebei University, Baoding, China,

^2^Hebei Provincial Key Laboratory of Skeletal Metabolic Physiology of Chronic Kidney Disease, Baoding, China

^3^The First Hospital of Hebei Medical University, Shijiazhuang, China.

^4^Affiliated Xingtai People's Hospital of Hebei Medial University, Xingtai, China.

***Correspondence:**

Haisong Zhang

[yxyzhanghaisong@hbu.edu.cn](mailto:yxyzhanghaisong@hbu.edu.cn)

**Supplementary Table S1: Primer sequences used for RT-qPCR**

| Gene | Forward primer (5’-3’) | Reverse primer (5’-3’) |
| --- | --- | --- |
| α-SMA | GTCCCAGACATCAGGGAGTAA | TCGGATACTTCAGCGTCAGGA |
| PYCARD | CTTGTCAGGGGATGAACTCAAAA | GCCATACGACTCCAGATAGTAGC |
| Foxp3 | CAGCAGGAGAAAGCGGATACC | GAAGACTTTGAGCAACCTGGAG |
| GAPDH | AGGTCGGTGTGAACGGATTTG | TGTAGACCATGTAGTTGAGGTCA |
| IFN-γ | CTGGAGGAACTGGCAAAAGGATGG | GACGCTTATGTTGTTGCTGATGGC |
| IL-1β | AAGCCTCGTGCTGTCGGACC | TGAGGCCCAAGGCCACAGGT |
| IL-6 | CACAGAGGATACCACTCCCAACA | TCCACGATTTCCCAGAGAACA |
| IL-10 | CCAGAGCCACATGCTCCTAGA | GGTCCTTTGTTTGAAAGAAAGTCTTC |
| IL-12 | CTAGACAAGGGCATGCTGGT | GCTTCTCCCACAGGAGGTTT |
| IL-17 | GTGTCTCTGATGCTGTTG | AACGGTTGAGGTAGTCTG |


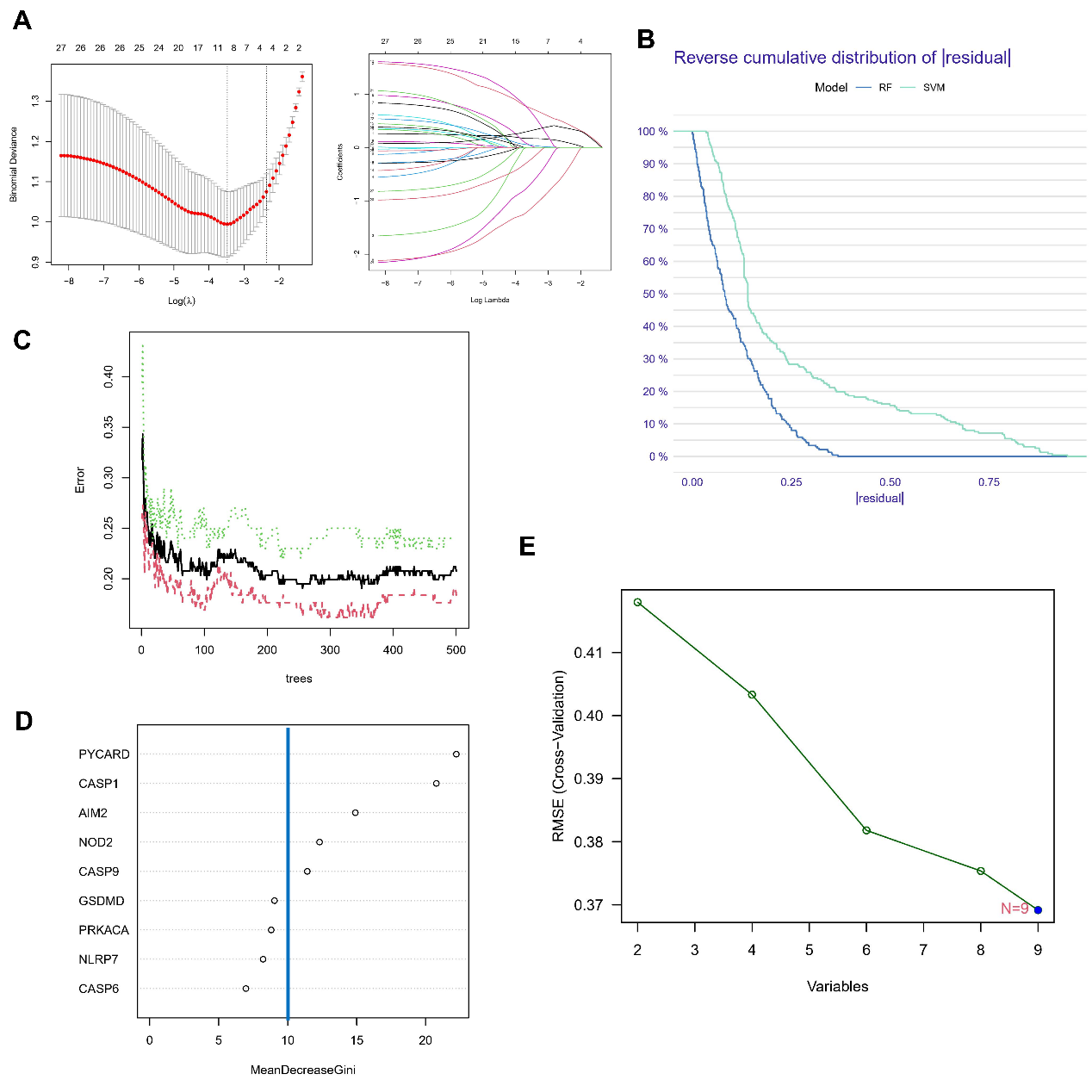


**Figure S1** Screening for hub PARs between normal and RF samples. (A) LASSO regression analysis of 27 regulators. (B) The reverse cumulative distribution of residuals in the random forest (RF) and SVM models was compared. (C-D) Genes with an importance of more than 10 in the random forest (RF) model and (E) SVM model.

**
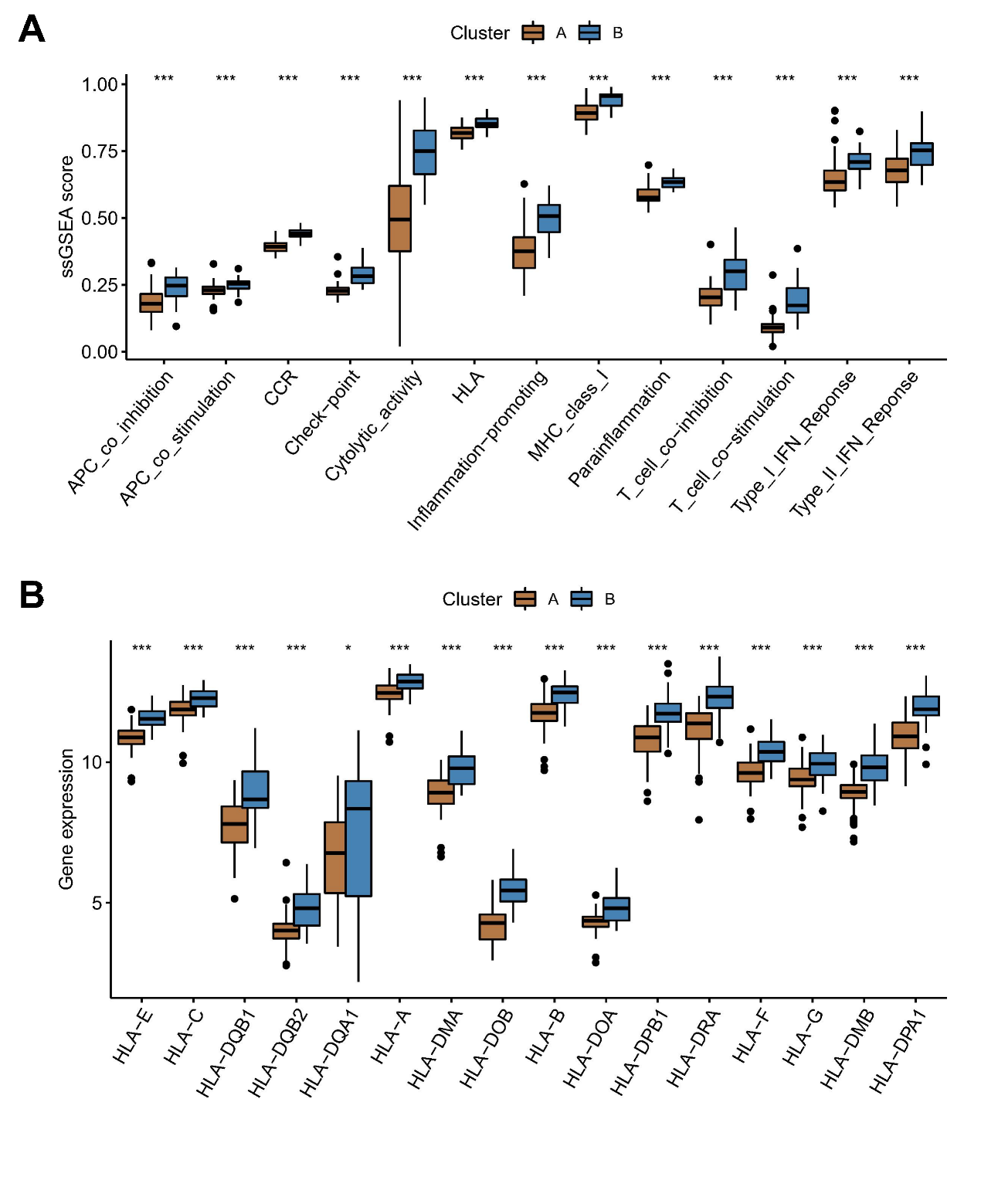
**

**Figure S2** Differences in immune microenvironmental features between different pyroptosis modification patterns. (A) Differences in immune function between the two pyroptosis modification patterns. (B) Differences in HLA gene expression between the two pyroptosis modification patterns.


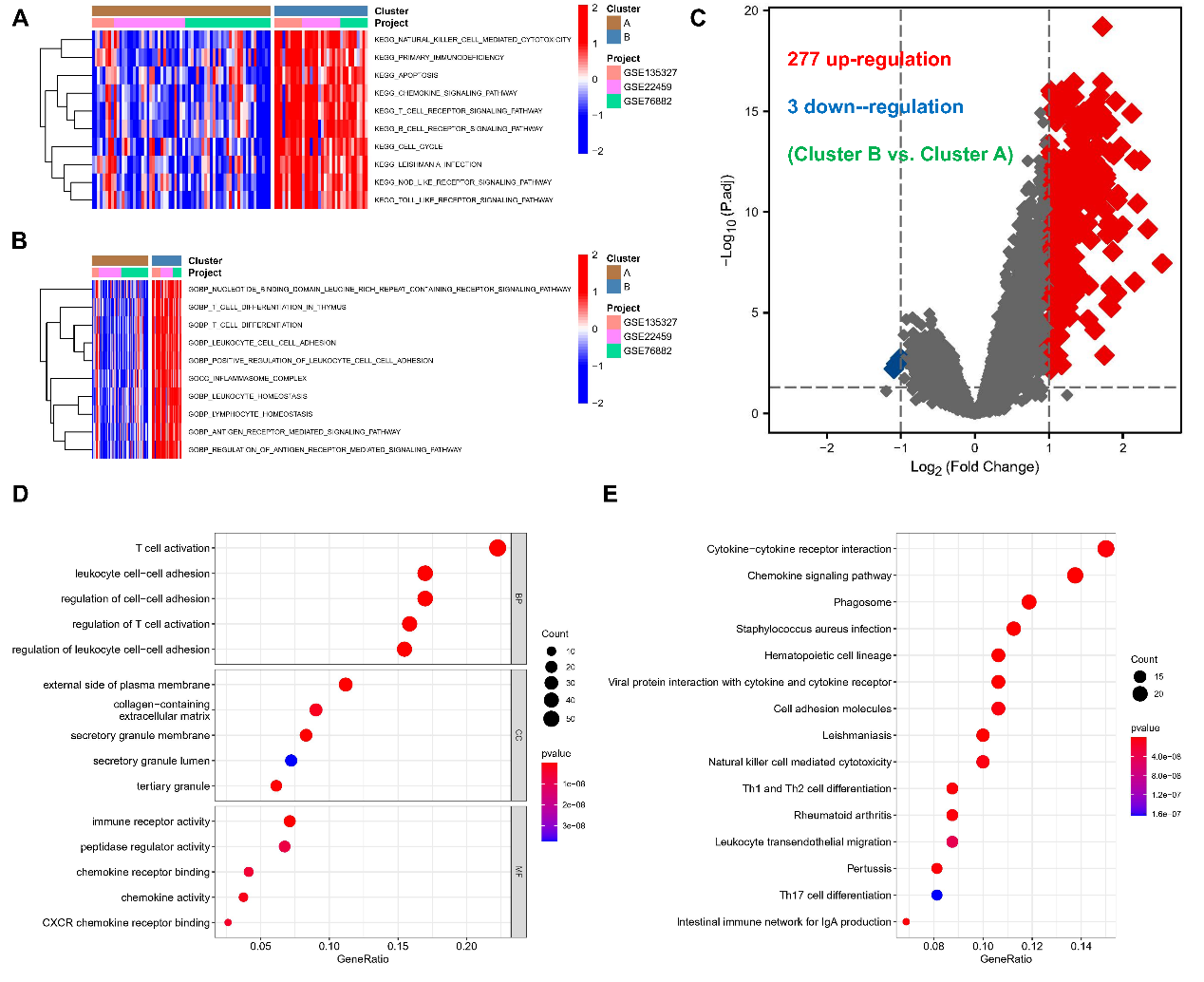


**Figure S3** Differences in biological characteristics between different pyroptosis modification patterns. (A) Differences in KEGG pathway enrichment scores between different pyroptosis modification patterns. (B) Differences in GO pathway enrichment scores between different pyroptosis modification patterns. (C) A total of 280 genes were related to different pyroptosis modification patterns. (D) GO functional enrichment analysis showed the correlations between genes and signaling pathways in immune cells between different pyroptosis modification patterns. (E) KEGG enrichment analysis revealed that genes related to pyroptosis were mainly involved in immune regulation processes.
